# Phylogeography and host specificity of *Pasteurellaceae* pathogenic to sea-farmed fish in the north-east Atlantic

**DOI:** 10.3389/fmicb.2023.1236290

**Published:** 2023-09-22

**Authors:** Snorre Gulla, Duncan J. Colquhoun, Anne Berit Olsen, Bjørn Spilsberg, Karin Lagesen, Caroline P. Åkesson, Sverri Strøm, Farah Manji, Thomas H. Birkbeck, Hanne K. Nilsen

**Affiliations:** ^1^Norwegian Veterinary Institute, Ås, Norway; ^2^Department of Biological Sciences, University of Bergen, Bergen, Norway; ^3^Norwegian Veterinary Institute, Bergen, Norway; ^4^Pharmaq Analytiq, Oslo, Norway; ^5^FoMAS – Fiskehelse og Miljø AS, Karmsund, Norway; ^6^Mowi Norway AS, Bergen, Norway; ^7^Division of Infection and Immunity, University of Glasgow, Glasgow, Scotland, United Kingdom

**Keywords:** Atlantic salmon (*Salmo salar*), lumpfish (*Cyclopterus lumpus*), pasteurellosis, *Pasteurellaceae*, *Pasteurella skyensis*, *“Pasteurella atlantica”*, *Phocoenobacter*, cetacean

## Abstract

The present study was undertaken to address the recent spate of pasteurellosis outbreaks among sea-farmed Atlantic salmon (*Salmo salar*) in Norway and Scotland, coinciding with sporadic disease episodes in lumpfish (*Cyclopterus lumpus*) used for delousing purposes in salmon farms. Genome assemblies from 86 bacterial isolates cultured from diseased salmon or lumpfish confirmed them all as *bona fide* members of the *Pasteurellaceae* family, with phylogenetic reconstruction dividing them into two distinct branches sharing <88% average nucleotide identity. These branches therefore constitute two separate species, namely *Pasteurella skyensis* and the as-yet invalidly named “*Pasteurella atlantica*”. Both species further stratify into multiple discrete genomovars (gv.) and/or lineages, each being nearly or fully exclusive to a particular host, geographic region, and/or time period. Pasteurellosis in lumpfish is, irrespective of spatiotemporal origin, linked almost exclusively to the highly conserved “*P. atlantica* gv. *cyclopteri*” (Pac). In contrast, pasteurellosis in Norwegian sea-farmed salmon, dominated since the late-1980s by “*P. atlantica* gv. *salmonicida*” (Pas), first saw three specific lineages (Pas-1, -2, and -3) causing separate, geographically restricted, and short-lived outbreaks, before a fourth (Pas-4) emerged recently and became more widely disseminated. A similar situation involving *P. skyensis* (Ps) has apparently been unfolding in Scottish salmon farming since the mid-1990s, where two historic (Ps-1 and -2) and one contemporary (Ps-3) lineages have been recorded. While the epidemiology underlying all these outbreaks/epizootics remains unclear, repeated detection of 16S rRNA gene amplicons very closely related to *P. skyensis* and “*P. atlantica*” from at least five cetacean species worldwide raises the question as to whether marine mammals may play a part, possibly as reservoirs. In fact, the close relationship between the studied isolates and *Phocoenobacter uteri* associated with harbor porpoise (*Phocoena phocoena*), and their relatively distant relationship with other members of the genus *Pasteurella*, suggests that both *P. skyensis* and “*P. atlantica*” should be moved to the genus *Phocoenobacter*.

## Introduction

Pasteurellosis has recently emerged as a disease of concern in Atlantic salmon (*Salmo salar*) sea-farmed in Norway and Scotland, with approximately 50 diagnosed cases registered annually in farms in south-western Norway from 2020 to 2022 (Soares et al., 2019; Axén et al., 2020; Legård and Strøm, 2020; Marine Scotland Directorate, 2022; Nilsen et al., 2022, 2023). These current epizootics contrast the situation of years past, following the first documented case in 1989 (then coined “Varracalbmi'), during which pasteurellosis in farmed salmon presented exlusively in the form of sporadic, localized, and short-lived outbreaks (Jones and Cox, 1999; Valheim et al., 2000; Birkbeck et al., 2002; Reid and Birkbeck, 2015). Pasteurellosis has also become established as a recurring and severe disease of farmed lumpfish (*Cyclopterus lumpus*) since their introduction to salmon farms for delousing purposes about a decade ago (Alarcón et al., 2016; Ellul et al., 2019).

The term “pasteurellosis” has for many years been used as a misnomer for systemic disease in fish caused by the bacterium *Photobacterium damselae* subsp. *piscicida* of the family *Vibrionaceae* (Gauthier et al., 1995), formerly known as *Pasteurella piscicida* (Janssen and Surgalla, 1968). Bacterial infections in fish linked to other “*Pasteurella*-like” organisms (Ajmal and Hobbs, 1967; Håstein and Bullock, 1976) have also been retrospectively confirmed as cases of mistaken identity (Paterson et al., 1980). True pasteurellosis in fish, however, caused by *bona fide* members of the family *Pasteurellaceae*, has to date exclusively been described (as outlined above) in sea-farmed Atlantic salmon and lumpfish in the north-eastern Atlantic region. Further use of the term “pasteurellosis” in the present study is thus exclusively with reference to such cases involving *bona fide Pasteurellaceae* species.

Although phenotypically highly similar, there appears to be considerable genetic and serological diversity amongst *Pasteurellaceae* isolated from farmed salmon and lumpfish (Reid and Birkbeck, 2015; Alarcón et al., 2016; Ellul et al., 2021). Further, while both field outbreaks and experimental challenge trials in lumpfish have often resulted in high mortality levels, pasteurellosis in salmon has been associated with variable but generally low to moderate mortality (Jones and Cox, 1999; Valheim et al., 2000; Alarcón et al., 2016; Ellul et al., 2019; Legård and Strøm, 2020; Sandlund et al., 2021). Whether these apparent differences reflect characteristics of the particular host species and/or bacterial strain involved, however, remains uncertain.

While only one bacterial species has been linked to pasteurellosis in Scottish salmon, i.e., *Pasteurella skyensis* (Birkbeck et al., 2002), most outbreaks in lumpfish and Norwegian salmon have been coupled to a distinct species recently proposed as “*Pasteurella atlantica*” (Alarcón et al., 2016; Gulla et al., 2020; Ellul et al., 2021; Nilsen et al., 2023). Although a degree of genetic heterogeneity amongst the various bacteria associated with pasteurellosis in fish is thus already recognized, their precise taxonomic position, genetic architecture, and population structure, remain largely undescribed. Their marine reservoirs are furthermore unknown, and the underlying causes of the current epizootic situations in Norway and Scotland have not been identified. Against this background, the present study was initiated to characterize, using whole genome sequencing, genetic diversity within a large collection of *Pasteurellaceae* isolated from diseased sea-farmed lumpfish and salmon.

## Materials and methods

### Bacterial isolates/strains, culture, and DNA extraction

The study involved 86 bacterial isolates/strains phenotypically consistent with *Pasteurella skyensis* or previously identified as “*Pasteurella skyensis*-like” or “*Pasteurella atlantica*” by Sanger sequencing of the 16S rRNA- and/or *rpoB* gene(s) (Alarcón et al., 2016). As shown in Table 1, all had been cultured from farmed Atlantic salmon (*n* = 65) or lumpfish (*n* = 21) in Norway (*n* = 73) or Scotland (*n* = 13) from 1991 to 2020, with the majority (*n* = 54) of the salmon isolates recovered from 2017 onwards. Regarding the Norwegian isolates specifically, these span 31 separate pasteurellosis cases in salmon across 26 different locations, and 17 lumpfish cases across 16 locations (not shown). Additionally, the type strain (NCTC 12872^T^) of the close *P. skyensis* relative *Phocoenobacter uteri* (Foster et al., 2000), recovered from harbor porpoise (*Phocoena phocoena*) in Scotland, was included as an outgroup, raising the total number of studied isolates/strains to 87.

**Table 1:** Metadata and genome accession nos. for the 87 fish- and porpoise-associated bacteria included in the present study.

**Isolate/strain**	Species^*^/genomovar	**Lineage**	**Geographic origin**	**Host**	**Year**	**Genome accession no**.
95A1^T^	*Pasteurella skyensis*	Ps-1	Scotland (Isle of Skye)	Atlantic salmon	1995	GCF_013377295.1
03D1	*Pasteurella skyensis*	Ps-1	Scotland (Ardnamurchan)	Atlantic salmon	2003	JASAYU000000000
VIO11850	*Pasteurella skyensis*	Ps-1	Norway (Vestland)	Atlantic salmon	2020	JASAVS000000000
VIO11851	*Pasteurella skyensis*	Ps-1	Norway (Vestland)	Atlantic salmon	2020	JASAVR000000000
98B1	*Pasteurella skyensis*	Ps-2	Scotland (Isle of Harris)	Atlantic salmon	1998	JASAYT000000000
01A1	*Pasteurella skyensis*	Ps-2	Scotland (Isle of Skye)	Atlantic salmon	2001	JASAYW000000000
TW342_17	*Pasteurella skyensis*	Ps-3	Scotland	Atlantic salmon	2017	JASAYL000000000
TW379_17	*Pasteurella skyensis*	Ps-3	Scotland	Atlantic salmon	2017	JASAYK000000000
TW34_18	*Pasteurella skyensis*	Ps-3	Scotland	Atlantic salmon	2017	JASAYM000000000
TW246_19	*Pasteurella skyensis*	Ps-3	Scotland	Atlantic salmon	2019	JASAYP000000000
TW260_19	*Pasteurella skyensis*	Ps-3	Scotland	Atlantic salmon	2019	JASAYO000000000
TW273_19	*Pasteurella skyensis*	Ps-3	Scotland	Atlantic salmon	2019	JASAYN000000000
TW16_20	*Pasteurella skyensis*	Ps-3	Scotland	Atlantic salmon	2019	JASAYQ000000000
VIB3689	“*Pasteurella atlantica* gv. *salmonicida*”	Pas-1	Norway (Troms)	Atlantic salmon	1991	JASAXA000000000
VIB3691	“*Pasteurella atlantica* gv. *salmonicida*”	Pas-1	Norway (Troms)	Atlantic salmon	1992	JASAWZ000000000
VIB3692	“*Pasteurella atlantica* gv. *salmonicida*”	Pas-1	Norway (Troms)	Atlantic salmon	1992	JASAWY000000000
VIB3131	“*Pasteurella atlantica* gv. *salmonicida*”	Pas-2	Norway (Vestland)	Atlantic salmon	1999	JASAXT000000000
VIB3693	“*Pasteurella atlantica* gv. *salmonicida*”	Pas-2	Norway (Vestland)	Atlantic salmon	1999	JASAWX000000000
VIB3695	“*Pasteurella atlantica* gv. *salmonicida*”	Pas-2	Norway (Vestland)	Atlantic salmon	2000	JASAWV000000000
VIB3694	“*Pasteurella atlantica* gv. *salmonicida*”	Pas-3	Norway (Vestland)	Atlantic salmon	2012	JASAWW000000000
VIB2635	“*Pasteurella atlantica* gv. *salmonicida*”	Pas-4	Norway (Vestland)	Atlantic salmon	2018	JASAYE000000000
VIB2680	“*Pasteurella atlantica* gv. *salmonicida*”	Pas-4	Norway (Vestland)	Atlantic salmon	2018	JASAYD000000000
VIB2717	“*Pasteurella atlantica* gv. *salmonicida*”	Pas-4	Norway (Vestland)	Lumpfish	2018	JASAYB000000000
VIB2829	“*Pasteurella atlantica* gv. *salmonicida*”	Pas-4	Norway (Vestland)	Atlantic salmon	2018	JASAYA000000000
VIB2913	“*Pasteurella atlantica* gv. *salmonicida*”	Pas-4	Norway (Rogaland)	Atlantic salmon	2018	JASAXZ000000000
VIB2921	“*Pasteurella atlantica* gv. *salmonicida*”	Pas-4	Norway (Rogaland)	Atlantic salmon	2018	JASAXY000000000
VIB2955	“*Pasteurella atlantica* gv. *salmonicida*”	Pas-4	Norway (Rogaland)	Atlantic salmon	2018	JASAXX000000000
VIB3014	“*Pasteurella atlantica* gv. *salmonicida*”	Pas-4	Norway (Vestland)	Atlantic salmon	2018	JASAXU000000000
VIB3781	“*Pasteurella atlantica* gv. *salmonicida*”	Pas-4	Norway (Rogaland)	Atlantic salmon	2018	JASAWI000000000
VIB3243	“*Pasteurella atlantica* gv. *salmonicida*”	Pas-4	Norway (Vestland)	Atlantic salmon	2019	JASAXS000000000
VIB3289	“*Pasteurella atlantica* gv. *salmonicida*”	Pas-4	Norway (Vestland)	Atlantic salmon	2019	JASAXQ000000000
VIB3296	“*Pasteurella atlantica* gv. *salmonicida*”	Pas-4	Norway (Vestland)	Atlantic salmon	2019	JASAXP000000000
VIB3305	“*Pasteurella atlantica* gv. *salmonicida*”	Pas-4	Norway (Vestland)	Atlantic salmon	2019	JASAXO000000000
VIB3308	“*Pasteurella atlantica* gv. *salmonicida*”	Pas-4	Norway (Vestland)	Atlantic salmon	2019	JASAXN000000000
VIB3309	“*Pasteurella atlantica* gv. *salmonicida*”	Pas-4	Norway (Vestland)	Atlantic salmon	2019	JASAXM000000000
VIB3490	“*Pasteurella atlantica* gv. *salmonicida*”	Pas-4	Norway (Vestland)	Lumpfish	2019	JASAXK000000000
VIB3506	“*Pasteurella atlantica* gv. *salmonicida*”	Pas-4	Norway (Vestland)	Atlantic salmon	2019	JASAXJ000000000
VIB3528	“*Pasteurella atlantica* gv. *salmonicida*”	Pas-4	Norway (Møre & Romsdal)	Atlantic salmon	2019	JASAXI000000000
VIB3624^T^	“*Pasteurella atlantica* gv. *salmonicida*”	Pas-4	Norway (Vestland)	Atlantic salmon	2019	JASAXH000000000
VIB3642	“*Pasteurella atlantica* gv. *salmonicida*”	Pas-4	Norway (Vestland)	Atlantic salmon	2019	JASAXG000000000
VIB3649	“*Pasteurella atlantica* gv. *salmonicida*”	Pas-4	Norway (Vestland)	Atlantic salmon	2019	JASAXF000000000
VIB3670	“*Pasteurella atlantica* gv. *salmonicida*”	Pas-4	Norway (Vestland)	Atlantic salmon	2019	JASAXE000000000
VIB3672	“*Pasteurella atlantica* gv. *salmonicida*”	Pas-4	Norway (Vestland)	Atlantic salmon	2019	JASAXD000000000
VIB3673	“*Pasteurella atlantica* gv. *salmonicida*”	Pas-4	Norway (Vestland)	Atlantic salmon	2019	JASAXC000000000
VIB3687	“*Pasteurella atlantica* gv. *salmonicida*”	Pas-4	Norway (Møre & Romsdal)	Atlantic salmon	2019	JASAXB000000000
VIB3782	“*Pasteurella atlantica* gv. *salmonicida*”	Pas-4	Norway (Rogaland)	Atlantic salmon	2019	JASAWH000000000
VIB3783	“*Pasteurella atlantica* gv. *salmonicida*”	Pas-4	Norway (Vestland)	Atlantic salmon	2019	JASAWG000000000
VIB3784	“*Pasteurella atlantica* gv. *salmonicida*”	Pas-4	Norway (Vestland)	Atlantic salmon	2019	JASAWF000000000
VIB3785	“*Pasteurella atlantica* gv. *salmonicida*”	Pas-4	Norway (Vestland)	Atlantic salmon	2019	JASAWE000000000
VIB3786	“*Pasteurella atlantica* gv. *salmonicida*”	Pas-4	Norway (Vestland)	Atlantic salmon	2019	JASAWD000000000
VIB3787	“*Pasteurella atlantica* gv. *salmonicida*”	Pas-4	Norway (Vestland)	Atlantic salmon	2019	JASAWC000000000
VIB3788	“*Pasteurella atlantica* gv. *salmonicida*”	Pas-4	Norway (Vestland)	Atlantic salmon	2019	JASAWB000000000
VIB3789	“*Pasteurella atlantica* gv. *salmonicida*”	Pas-4	Norway (Vestland)	Atlantic salmon	2019	JASAWA000000000
VIB3790	“*Pasteurella atlantica* gv. *salmonicida*”	Pas-4	Norway (Vestland)	Atlantic salmon	2019	JASAVZ000000000
VIB3791	“*Pasteurella atlantica* gv. *salmonicida*”	Pas-4	Norway (Vestland)	Atlantic salmon	2019	JASAVY000000000
VIB3707	“*Pasteurella atlantica* gv. *salmonicida*”	Pas-4	Norway (Vestland)	Atlantic salmon	2020	JASAWT000000000
VIB3708	“*Pasteurella atlantica* gv. *salmonicida*”	Pas-4	Norway (Vestland)	Atlantic salmon	2020	JASAWS000000000
VIB3709	“*Pasteurella atlantica* gv. *salmonicida*”	Pas-4	Norway (Vestland)	Atlantic salmon	2020	JASAWR000000000
VIB3710	“*Pasteurella atlantica* gv. *salmonicida*”	Pas-4	Norway (Vestland)	Atlantic salmon	2020	JASAWQ000000000
VIB3711	“*Pasteurella atlantica* gv. *salmonicida*”	Pas-4	Norway (Vestland)	Atlantic salmon	2020	JASAWP000000000
VIB3760	“*Pasteurella atlantica* gv. *salmonicida*”	Pas-4	Norway (Vestland)	Atlantic salmon	2020	JASAWO000000000
VIB3763	“*Pasteurella atlantica* gv. *salmonicida*”	Pas-4	Norway (Vestland)	Atlantic salmon	2020	JASAWN000000000
VIB3764	“*Pasteurella atlantica* gv. *salmonicida*”	Pas-4	Norway (Vestland)	Atlantic salmon	2020	JASAWM000000000
VIB3766	“*Pasteurella atlantica* gv. *salmonicida*”	Pas-4	Norway (Vestland)	Atlantic salmon	2020	JASAWL000000000
VIB3767	“*Pasteurella atlantica* gv. *salmonicida*”	Pas-4	Norway (Vestland)	Atlantic salmon	2020	JASAWK000000000
VIB3770	“*Pasteurella atlantica* gv. *salmonicida*”	Pas-4	Norway (Vestland)	Atlantic salmon	2020	JASAWJ000000000
VIB3834	“*Pasteurella atlantica* gv. *salmonicida*”	Pas-4	Norway (Vestland)	Atlantic salmon	2020	JASAVU000000000
VIO3648	“*Pasteurella atlantica* gv. *cyclopteri*”	Pac	Norway (Troms)	Lumpfish	1996	JASAVQ000000000
VIB164	“*Pasteurella atlantica* gv. *cyclopteri*”	Pac	Norway (Rogaland)	Lumpfish	2012	JASAYG000000000
VIB3802	“*Pasteurella atlantica* gv. *cyclopteri*”	Pac	Norway (Nordland)	Lumpfish	2013	JASAVW000000000
VIB3804	“*Pasteurella atlantica* gv. *cyclopteri*”	Pac	Norway (Nordland)	Lumpfish	2013	JASAVV000000000
VIB543	“*Pasteurella atlantica* gv. *cyclopteri*”	Pac	Norway (Rogaland)	Lumpfish	2013	JASAVT000000000
VIO9100	“*Pasteurella atlantica* gv. *cyclopteri*”	Pac	Norway (Møre & Romsdal)	Lumpfish	2013	GCF_018343795.1
VIB1234	“*Pasteurella atlantica* gv. *cyclopteri*”	Pac	Norway (Vestland)	Lumpfish	2015	JASAYJ000000000
VIB1365	“*Pasteurella atlantica* gv. *cyclopteri*”	Pac	Norway (Vestland)	Lumpfish	2016	JASAYI000000000
VIB1366	“*Pasteurella atlantica* gv. *cyclopteri*”	Pac	Norway (Vestland)	Lumpfish	2016	JASAYH000000000
VIB1926	“*Pasteurella atlantica* gv. *cyclopteri*”	Pac	Norway (Vestland)	Lumpfish	2017	JASAYF000000000
VIB3801	“*Pasteurella atlantica* gv. *cyclopteri*”	Pac	Norway (Troms)	Lumpfish	2017	JASAVX000000000
TW138_17	“*Pasteurella atlantica* gv. *cyclopteri*”	Pac	Scotland	Lumpfish	2017	JASAYS000000000
TW141_17	“*Pasteurella atlantica* gv. *cyclopteri*”	Pac	Scotland	Lumpfish	2017	JASAYR000000000
VIB2702	“*Pasteurella atlantica* gv. *cyclopteri*”	Pac	Norway (Vestland)	Lumpfish	2018	JASAYC000000000
VIB2993	“*Pasteurella atlantica* gv. *cyclopteri*”	Pac	Norway (Vestland)	Lumpfish	2018	JASAXW000000000
VIB3004	“*Pasteurella atlantica* gv. *cyclopteri*”	Pac	Norway (Vestland)	Lumpfish	2018	JASAXV000000000
VIB3273	“*Pasteurella atlantica* gv. *cyclopteri*”	Pac	Norway (Rogaland)	Lumpfish	2019	JASAXR000000000
VIB3468	“*Pasteurella atlantica* gv. *cyclopteri*”	Pac	Norway (Vestland)	Lumpfish	2019	JASAXL000000000
VIB3703	“*Pasteurella atlantica* gv. *cyclopteri*”	Pac	Norway (Vestland)	Lumpfish	2020	JASAWU000000000
NCTC12872^T^	*Phocoenobacter uteri*		Scotland (Inverness)	Harbor porpoise	1993	GCF_900454895.1

^*^“*Pasteurella atlantica*”, with VIB-3624^T^ as the tentative type strain (Nilsen et al., unpublished), is not yet a validly named species, but both ANI comparisons and phylogenetic recreation certify its distinction from *Pasteurella skyensis* (see “Discussion”).

For isolates sequenced in-house (see below), cryopreserved cultures were first spread onto 5% bovine blood agar supplemented with NaCl to a final concentration of 2.0%, and incubated at 22°C for 2–4 days prior to DNA extraction using the QIAamp DNA kit and QIAamp DNA Accessory kit Set A (Qiagen) according to the manufacturer's instructions.

### Whole genome sequencing, assembly, and annotation

In-house sequencing of 84 of the studied isolates was performed using an Illumina MiSeq platform. Libraries were prepared using the NexteraFlex kit (Illumina) according to the manufacturer's recommendations, with sequencing on a V3 flow cell outputting 300 bp paired end reads. Subsequent quality assessment, trimming, and assembly was done using the NVI Bifrost pipeline v1.2 (Lagesen, 2020). This involved (in chronological order) quality assessment with FastQC v0.11.9 (Andrews, 2010) and MultiQC v1.9 (Ewels et al., 2016), PhiX stripping with BBDuk (BBMap v38.76; Bushnell, 2014), trimming with Trimmomatic v0.39 (Bolger et al., 2014), assembly with Spades v3.14 (Bankevich et al., 2012), read coverage mapping with BWA-MEM v0.7.17 (Li, 2013), assembly polishing with Pilon v1.23 (Walker et al., 2014), and assembly evaluation with Quast v5.0.2 (Gurevich et al., 2013). While Spades was run under the—careful option, all other programs were run on default settings, with the complete genome of *P. skyensis* type strain 95A1^T^ used as a reference whenever needed. Finally, contigs shorter than 501 bp and/or with a coverage below 5 were removed from all assemblies. In addition to the 84 genome assemblies thus generated in-house, publicly available assemblies were downloaded from the NCBI RefSeq repository for *P. skyensis* strain 95A1^T^, “*P. atlantica*” strain VIO9100 and *Ph. uteri* strain NCTC 12872^T^.

The ncbi-genome-download tool (github.com/kblin/ncbi-genome-download) was used on June 17th 2020 to download all annotated genomes in the *Pasteurellaceae* family available through the NCBI RefSeq database. These were subsequently concatenated into a single file before being converted to fasta-format using the prokka-genbank_to_fasta_db function in Prokka (Seemann, 2014). Prokka v1.14.5, with this file as input under the—proteins flag, was then employed for (re)annotation of all 87 genome assemblies.

### Comparative genomics

Based on the 87 annotated assemblies, pan- and core-genomes were inferred by use of Roary v3.13 (Page et al., 2015) with the minimum protein identity set to 85% (or 95% for con specific sub-selections; see Results), and with the core genes thus identified subsequently concatenated and aligned in PRANK (Löytynoja, 2014). This alignment was then used as input for maximum likelihood (ML) phylogenetic reconstruction in IQ-TREE v1.6.12 (Nguyen et al., 2015), employing ModelFinder (Kalyaanamoorthy et al., 2017) across all GTR, HKY, and SYM models, and estimating branch support with 1000 ultrafast bootstrap replicates (Minh et al., 2013). The resulting tree and associated metadata was uploaded to Microreact v232 (Argimón et al., 2016) for visualization purposes.

A pairwise single nucleotide polymorphism (SNP) distance matrix, based on the same concatenated core gene alignment, was generated using snp-dists v0.6.3 (github.com/tseemann/snp-dists) on default settings. FastANI v1.3 (Jain et al., 2018), with—fragLen set to 500 and—minFraction set to 0.1, was used to generate a pairwise average nucleotide identity (ANI) matrix for the 87 genome assemblies. The two (SNP and ANI) distance matrices were then merged into a single square matrix (Supplementary Table S1).

### ANI comparison with other *Pasteurellaceae*

Type strain genome assemblies from 12 selected *sensu stricto* (Christensen and Bisgaard, 2018; Kirchner et al., 2019; Zheng et al., 2020) species belonging to the *Pasteurellaceae* genera *Pasteurella* (*P. dagmatis* and *P. multocida* subspp. *multocida, gallicida*, and *septica*), *Haemophilus* (*H. aegyptius, H. haemolyticus, H. influenza*, and *H. seminalis*), and *Actinobacillus* (*A. capsulatus, A. pleuropneumoniae, A. suis*, and *A. vicugnae*) were downloaded from the NCBI GenBank repository (see Supplementary Table S2 for genome accession nos.). Then, again using FastANI v1.3 (Jain et al., 2018) with—fragLen set to 500 and—minFraction set to 0.1, a second ANI matrix was generated for pairwise comparison of these 12 genomes in addition to single representative genomes of *P. skyensis* (95A1^T^), “*P. atlantica*” (VIO9100) and *Ph. uteri* (NCTC 12872^T^). This matrix was subsequently used directly as input for neighbor joining (NJ) in R v4.1.2 with the package APE v5.6.1 (Paradis et al., 2004), before a NJ tree was generated with ggtree v3.2.1 (Yu et al., 2017).

### Comparative 16s rRNA gene analyses

By use of a previously published 16S rRNA gene sequence from *P. skyensis* strain 95A1^T^ (accession no. AJ243202) as query in the NCBI online blastn tool, all unique sequence hits (i.e. disregarding duplicates) displaying ≥ 88% coverage and ≥ 96% identity discovered in the NCBI Nucleotide database were downloaded. In addition, reference 16S rRNA gene sequences from the 12 *Pasteurellaceae* type strains mentioned above were also downloaded. Following trimming of the compiled sequences to adjust for any lacking flank coverage, an alignment was produced in ClustalX v2.1 (Larkin et al., 2007) with default settings. This was then used as input in PhyML v3.0 (Guindon et al., 2010) for generation of a ML phylogenetic tree, with model selection by SMS (Lefort et al., 2017) and branch support estimation by the aLRT SH-like method (Anisimova and Gascuel, 2006).

Stand-alone queries using the aforementioned *P. skyensis* 95A1^T^ 16S rRNA sequence were also performed with the NCBI online blastn tool toward sequences deposited in the Sequence Read Archive (SRA) from two specific studies (accession nos. SRP072736 and SRP163436). These datasets derive from two amplicon-based microbiome surveys involving harbor porpoise, common dolphin (*Delphinus delphis*), and/or striped dolphin (*Stenella coeruleoalba*) stranded on the Iberian coast, with both reporting a predominance of *Phocoenobacter*-related sequences amongst oral samples (Godoy-Vitorino et al., 2017; Soares-Castro et al., 2019).

## Results

### Genome assemblies

The 84 genome assemblies generated in-house are composed of 30–95 (avg. 62) contigs, with N50-values ranging between 59 and 249 (avg. 106) kbp. Their sizes range between 2.12 and 2.29 (avg. 2.20) Mbp, with GC-content percentages of 34.20–34.96 (avg. 34.37). All 84 assemblies were uploaded to NCBI under BioProject accession no. PRJNA727067 (see Table 1 for genome accession nos.).

### Phylogenetic recreation

**Figure 1:**
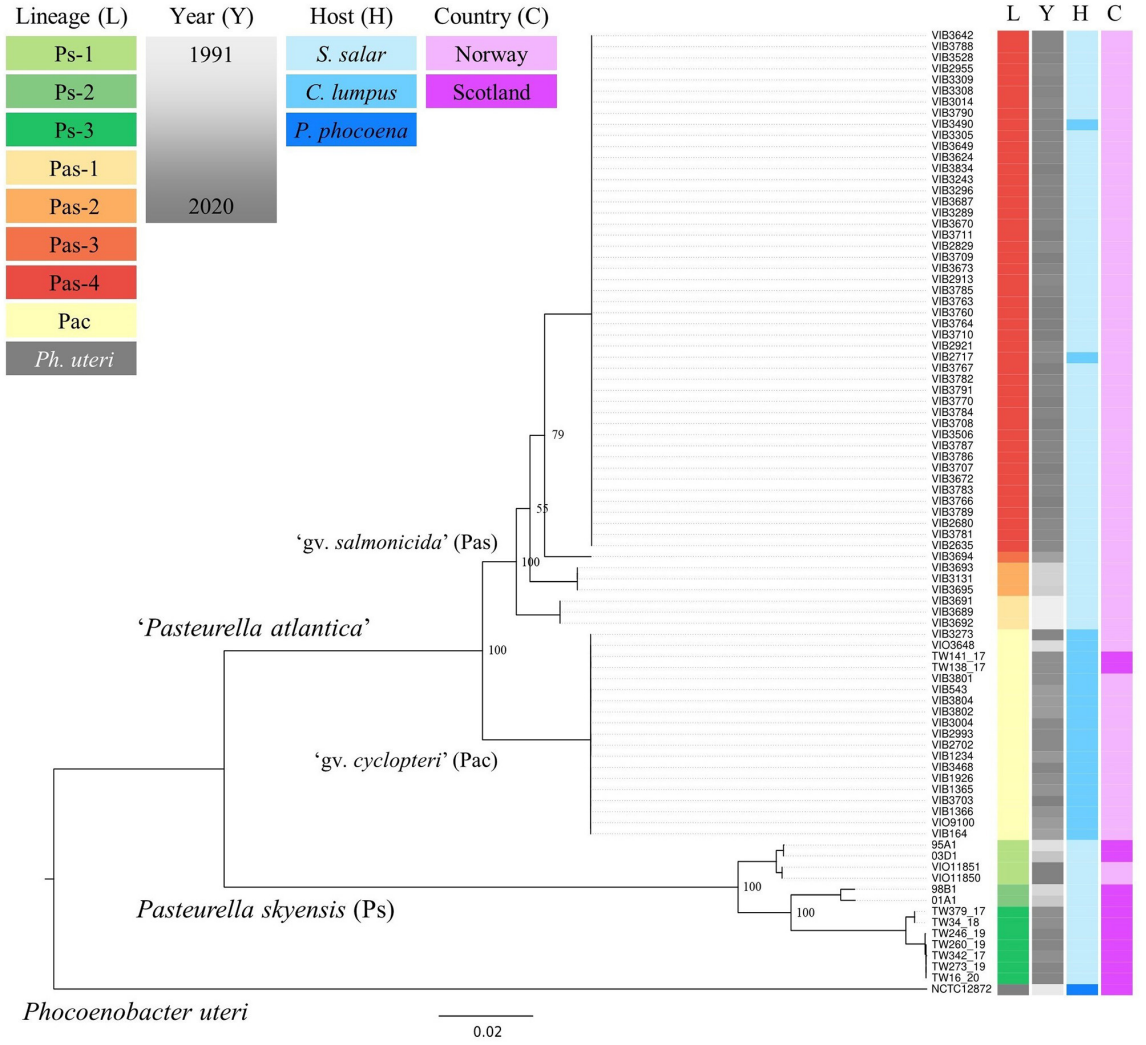
ML core gene phylogenetic reconstruction for the 87 fish- and porpoise-associated bacterial genomes studied. Colored columns on the right indicate lineage (L), year (Y), host (H), and country (C) affiliations – see upper left legends. The tree was midpoint-rooted for illustrative purposes, and bootstrap support values are shown for bifurcations separating the named lineages. The scale bar refers to nucleotide substitutions per site. Metadata annotations were added using Microreact (Argimón et al., 2016).

Within an 85% protein identity threshold, a pan-genome consisting of 6,037 genes was identified by Roary across all the 87 *Pasteurellaceae* genomes from fish and porpoise studied (i.e., including also the three sourced from the NCBI RefSeq repository), with 691 core gene homologs found present in all. The 700 659 bp concatenated core gene alignment produced from these contains 148 336 SNPs, 103 975 of which are parsimony informative. The inferred ML phylogeny (built under the GTR+F+R7 model, as favored by ModelFinder) reveals the existence of a deep divide, separating the 86 fish-associated genomes into two main branches (Figure 1). One branch comprises all previously identified isolates/strains of the validly published *P. skyensis*, while the other constitutes the as-yet non-validated taxon “*P. atlantica*”, with both branches stratifying further into multiple distinct genomovars/lineages. *P. skyensis* (Ps) as such divides into three main lineages (Ps-1, -2, and -3), whereas “*P. atlantica*” bifurcates into two sub-branches, denoted here as genomovars “*cyclopteri*” (Pac) and “*salmonicida*” (Pas), reflective of their primary host associations. The latter then splits into a further four lineages (Pas-1, -2, -3, and -4), although low bootstrap values associated with two of these divisions render the exact order in which they took place uncertain.

Re-runs of Roary (within a 95% protein identity threshold) for each of the two main fish-associated branches separately identified pan-/core-genomes consisting of 3513/1393 genes in *P. skyensis* and 3476/1253 genes in “*P. atlantica*”

**Figure 2:**
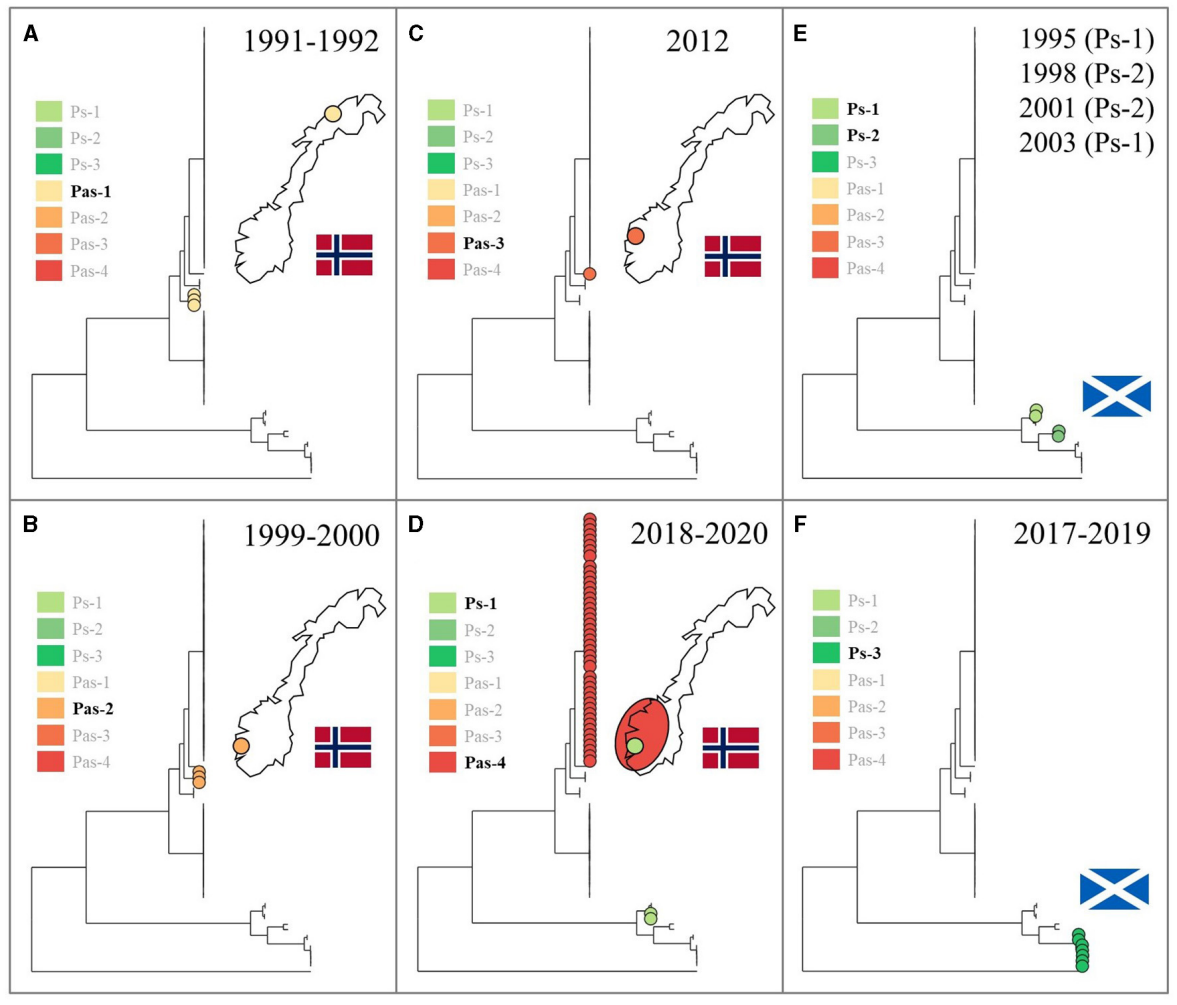
Using the same phylogenetic tree as in Figure 1, chronological panels show the known spatiotemporal distribution, respectively in Norway **(A–D)** and Scotland **(E, F)**, for each of the salmon-associated “*Pasteurella atlantica* gv. *salmonicida*” (Pas) and *Pasteurella skyensis* (Ps) lineages. Leftmost insets highlight (with non-translucent text) the lineage(s) documented within each time period and geographic region. Fewer details were available regarding the origins of Scottish isolates.

Upon relating the respective taxonomic strata (species, genomovars, lineages) denoted in Figure 1 to the underlying metadata, it becomes evident that each of them associates strongly with a specific host species, country, and/or time period (see also Table 1). The lumpfish-specific “*P. atlantica* gv. *cyclopteri*” dominates in this fish species irrespective of spatiotemporal origin. Contrarily, the four “*P. atlantica* gv. *salmonicida*” lineages have been found only in Norway, where they dominate almost exclusively in farmed salmon, while the three *P. skyensis* lineages account for all Scottish salmon cases (Figure 2). Notably, while each of the “historic” salmon-associated lineages documented in Norway (Pas-1, -2, and -3) have only been found in individual fjords over relatively short time spans, the contemporary Pas-4 lineage has become much more widely dispersed, and currently affects farms all along the south-western coastline. Similar interpretations could not be performed for Scottish salmon isolates due to their limited number and less accurate geographic annotations.

### Pairwise SNP and ANI similarity matrices

**Figure 3:**
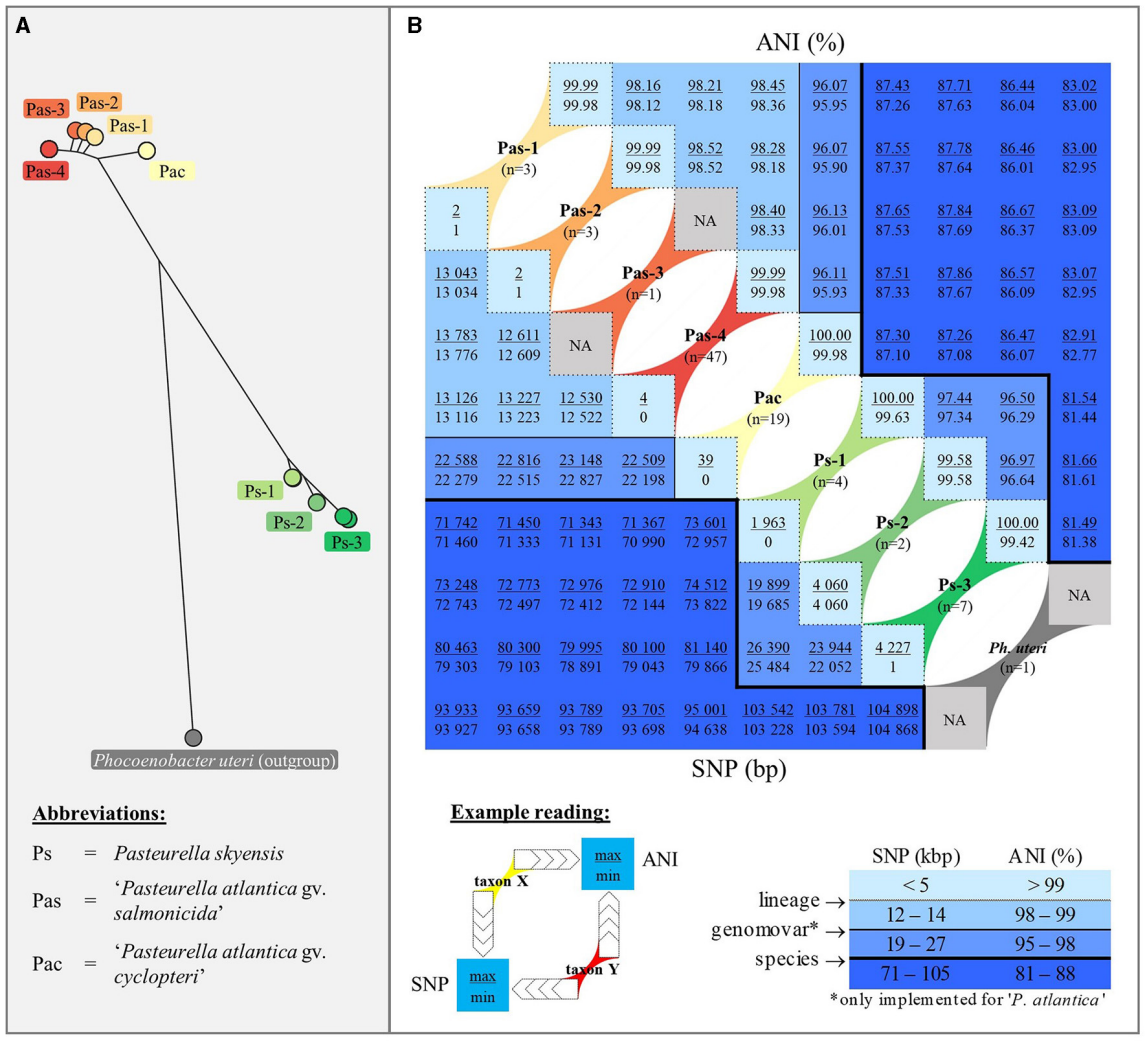
Panel **(A)**: For reference, the same phylogeny as displayed in Figure 1 is shown here in radial layout, with separate colors highlighting the three *P. skyensis* and five “*P. atlantica*” lineages, as well as the *Ph. uteri* outgroup. Panel **(B)**: Combined square matrix summarizing pairwise genetic similarities (ANI – upper right triangular; number of SNPs – lower left triangular) among and between genomes belonging to each of the nine taxa highlighted in panel A, using identical color coding and (for SNP) based on the same concatenated core gene alignment. Below the matrix features an example reading of max/min distances between hypothetical taxa X and Y, alongside a table illustrating the darker shades of blue used to reflect decreasing genetic similarities. This table also shows applied breaking points for lineage, genomovar, and species separation, which recur as border lines within the matrix. For full (per-genome-pair) matrices, see Supplementary Table S1.

Separation of the respective *P. skyensis* and “*P. atlantica*” genomovars/lineages named according to the SNP-based core gene phylogeny is unambiguously supported by pairwise ANI comparisons, as shown opposite SNP distances in Figure 3. Based on the genomes examined here, *P. skyensis* and “*P. atlantica*” display ~86–88% ANI similarity when compared against each other and ~81–83% when compared to the *Ph. uteri* outgroup. ANI similarities within the two species range ≥ 96.29% for *P. skyensis* and ≥ 95.90% for “*P. atlantica*”, while it ranges ≥ 99.42% within each of the seven designated lineages. See Supplementary Table S1 for full (per-genome-pair) ANI and SNP distance matrices.

### ANI comparison with other *Pasteurellaceae*

**Figure 4:**
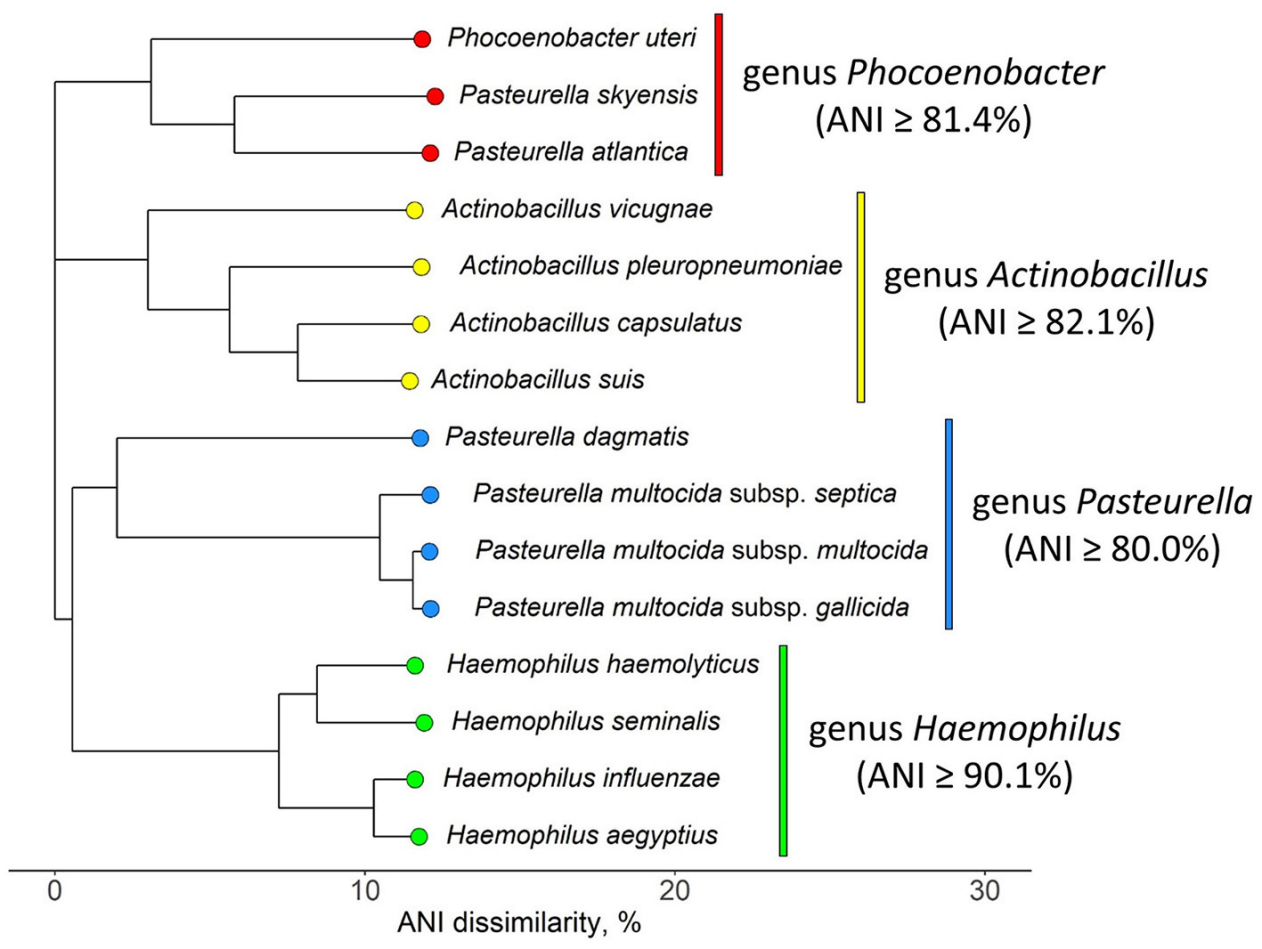
NJ tree displaying ANI dissimilarities (in percent) between selected *sensu stricto* members of the *Pasteurellaceae* genera *Actinobacillus, Pasteurella*, and *Haemophilus*, alongside *Pasteurella skyensis*, “*Pasteurella atlantica”*, and *Phocoenobacter uteri*. The three latter species form a distinct monophyletic branch (genus *Phocoenobacter*) comparable to those branches constituting the other three genera. See Supplementary Table S2 for strain identifiers, genome accession nos., and ANI matrix.

The NJ tree inferred from pairwise ANI comparison involving representative *P. skyensis*, “*P. atlantica*”, and *Ph. uteri* genomes, in addition to selected *sensu stricto* members of the *Pasteurella, Haemophilus*, and *Actinobacillus* genera, is shown in Figure 4 (matrix in Supplementary Table S2). While the *Haemophilus* and *Actinobacillus* genera display distinct, monophyletic branches, it appears clear that *Pasteurella skyensis* and “*Pasteurella atlantica*” do not represent *sensu stricto* members of the genus *Pasteurella*, as they instead form a separate fourth monophyletic branch together with *Phocoenobacter uteri*, the single recognized member of its genus.

### 16s rRNA gene phylogeny vs. host origin

**Figure 5:**
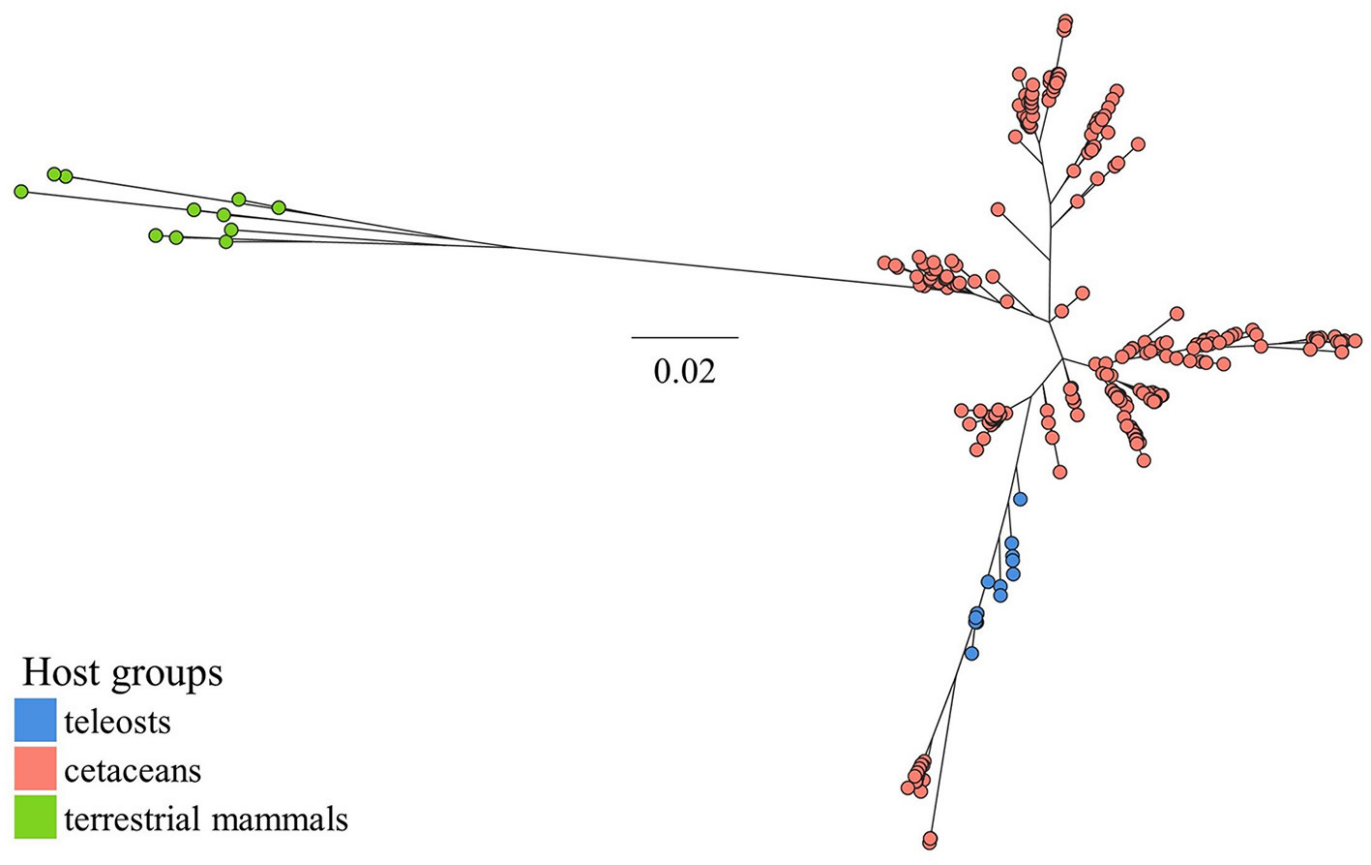
ML phylogeny inferred from a 1390 bp alignment of 349 *Pasteurellaceae* 16S rRNA gene sequences sampled from various host species groups (see lower left legend). Bacterial taxa represented per host group are, for teleosts, *Pasteurella skyensis* and “*Pasteurella atlantica”*; for cetaceans *Phocoenobacter uteri* and misc. unspecified taxa; and for terrestrial mammals *Pasteurella* spp., *Haemophilus* spp. and *Actinobacillus* spp. The scale bar refers to nucleotide substitutions per site. See Supplementary Figure S1 for sequence accession nos.

Blastn searches into the NCBI nucleotide database produced 337 unique (duplicates exempt) 16S rRNA gene sequences with ≥ 96% similarity (and ≥ 88% coverage) toward the *P. skyensis* query sequence. While 16 of these matches belong to verified strains of *P. skyensis*, “*P. atlantica*”, or *Ph. uteri*, the remaining 321 derive exclusively from uncultivated samples collected from the gastrointestinal tract of bottlenose dolphins (*Tursiops truncates*) (Bik et al., 2016) or from the skin of humpback whale (*Megaptera novaeangliae*) (Apprill et al., 2011). An additional 12 reference sequences were collected from the aforementioned *Pasteurella, Haemophilus*, and *Actinobacillus* type strains. From the 349 sequences thus compiled resulted a 1390 bp alignment which was used as input for the phylogenetic reconstruction shown in Figure 5 (for sequence accession nos., see Supplementary Figure S1).

Separate *P. skyensis* 16S rRNA queries made toward specific SRA amplicon datasets produced multiple (in most cases hundreds or thousands) 100% hits among all 38 oral samples from harbor porpoise, common dolphin, and striped dolphin deposited by Soares-Castro et al. (2019). Similarly, more than 4000 hits with 100% sequence identity were identified across the eight bodily niches of a single striped dolphin sampled by Godoy-Vitorino et al. (2017), with > 99% of these hits originating from oral samples. As both studies were limited to the V3- and/or V4-region of the 16S rRNA gene, however, these sequences were not included in the phylogenetic analysis mentioned above.

## Discussion

In the present study, we confirm that *Pasteurella skyensis* and “*Pasteurella atlantica*” isolated from diseased, sea-farmed Atlantic salmon and lumpfish in Norway and Scotland represent two relatively closely related yet discrete bacterial taxa. They are separated by ANI values between ~86 and 88%, i.e. well below the generally accepted threshold for bacterial species separation at ~95–96% (Richter and Rosselló-Móra, 2009), and should therefore be regarded as separate species. A considerable degree of intra-species diversity is also observed within each of them, with individual lineages showing a strong correlation to host and/or spatiotemporal origin. The common occurrence of closely related bacteria on mucosal surfaces, particularly oral, of various cetacean species may indicate marine mammals as a possible reservoir.

While our findings verify that all the *P. skyensis* and “*P. atlantica*” isolates investigated represent *bona fide* members of the family *Pasteurellaceae*, further confirmation of their close relationship with *Phocoenobacter uteri* does question their taxonomic placement within the genus *Pasteurella*. *P. skyensis* was originally placed within this genus based on phenotypical characterization and 16S rRNA gene sequence similarity with the invalidly published bacterium “*Pasteurella phocoenarum*” NCTC 12872 (Birkbeck et al., 2002), isolated from the uterus of a harbor porpoise. As strain NCTC 12872^T^ had in fact been validly published 2 years prior under the name *Phocoenobacter uteri* (Foster et al., 2000), the initial association of *P. skyensis* with the genus *Pasteurella* may be considered imprecise.

Here, we find that *P. skyensis* and “*P. atlantica*” are separate from *Ph. uteri* within ANI similarities of ~81–83% (Figure 3), which is significantly above the mean genus demarcation boundary of 73.98% estimated by Barco et al. (2020) across 858 bacterial and archaeal genera. Moreover, the ANI-based NJ tree in Figure 4 shows that these three taxa together form a distinct monophyletic branch similar to those formed by other *sensu stricto* representatives (Christensen and Bisgaard, 2018; Kirchner et al., 2019; Zheng et al., 2020) of various *Pasteurellaceae* genera, including *Pasteurella*. Taken together, these results present a compelling case for the reallocation of *Pasteurella skyensis* and “*Pasteurella atlantica*” to the genus *Phocoenobacter*, although further work will be necessary to clarify and validly publish their precise taxonomic position. Pending such validation, the currently recognized nomenclature was retained for the purpose of communicating our findings from the present study.

Irrespective of their final taxonomic placement, however, phylogenetic reconstruction reveals the further stratification of both *P. skyensis* and “*P. atlantica*” into discrete genomovars and/or lineages relating strongly to their temporal-, geographic-, and/or host origins (Figure 1). Amongst these, “*P. atlantica* gv. *cyclopteri*” (Pac) appears to be specifically linked to lumpfish, having been responsible for a relatively common and serious disease in this fish species in Norwegian and Scottish aquaculture, particularly over the last decade. Contrarily, all of the remaining “*P. atlantica* gv. *salmonicida*” (Pas-1, -2, -3, and -4) and *P. skyensis* (Ps-1, -2, and -3) lineages derive almost exclusively from farmed Atlantic salmon in Norway and Scotland respectively, with the only non-salmon hosts represented being two lumpfish stocked together with salmon suffering a pasteurellosis outbreak involving Pas-4.

Amongst the salmon-associated lineages documented prior to 2017 (i.e., Pas-1, -2, and -3, and Ps-1 and -2), all appear associated with relatively short-lived, local outbreaks (Figure 2), although Ps-1, originally detected in Scotland in 1995 and 2003 (Birkbeck et al., 2002; Reid and Birkbeck, 2015), was subsequently identified at two neighboring Norwegian salmon farms in 2020 (Strøm and Nilsen, 2021). This is in contrast to the contemporary salmon epizootics involving Pas-4 in Norway and Ps-3 in Scotland, which both appear to have established a better footing in aquaculture compared to their predecessors, with Pas-4 in particular now widely dispersed in south-western Norway. While the high degree of genetic conservation within this latter lineage has not yet allowed precise identification of its geographical origin(s) or dispersion patterns, all initial (2018) detections originate from the southernmost part of the affected area (not shown), making this the likely “epicenter”.

Taken together, the results generated seem to reflect the endemic presence in the North-East Atlantic of an evolutionary conserved and specialized lumpfish pathogen, i.e. “*P. atlantica* gv. *cyclopteri*” (Pac), alongside multiple “*P. atlantica* gv. *salmonicida*” (Pas-1, -2, -3, and -4) and *P. skyensis* (Ps-1, -2, and -3) lineages with a predilection for salmon (Figure 1). Although two lineages (Pac and Ps-1) include isolates recovered over spans of 25+ years, they each appear to have undergone relatively modest genetic diversification during this time. This is reflected by their *intra*-lineage ANI values ≥ 99.63% (Figure 3). In comparison, the *inter*-lineage ANI values (≤ 98.52%) separating all salmon-associated lineages point to a considerably higher degree of genetic diversification between these. Assuming similar mutation rates, this in turn suggests that all of them likely diverged from one another significantly prior to the arrival of salmon aquaculture. From this follows the probable existence of one or more hitherto unidentified marine reservoir(s), having continuously maintained each of the salmon-associated *P. skyensis* and “*P. atlantica* gv. *salmonicida*” lineages described here. We thus speculate that the typically abrupt, localized, and transient nature of all but the most recent salmon pasteurellosis episodes (Figure 2) could be indicative of sporadic infection “spillover” from such an unknown reservoir.

Notable in this regard is the emergence in the early 2000s of *Streptococcus phocae*, previously associated primarily with pinnipeds, as a pathogen of concern in Chilean salmon farming, which raised suspicions of an initial introduction via host spillover having preceded onward spread via the movement of fish (Romalde et al., 2008). Unwanted close interactions between European farmed salmon and predatory mammalian wildlife are not unheard of Quick et al. (2004) and BBC News (2021), and a putative link has previously been drawn between salmon pasteurellosis and marine mammals (Reid and Birkbeck, 2015). Here, we further substantiate this connection by documenting sequences near identical to those found in *P. skyensis* and “*P. atlantica*” as dominant constituents of 16S rRNA amplicon datasets generated from various marine cetacean species. Phylogenetic reconstruction based on a 1390 bp 16S rRNA gene alignment does not clearly distinguish between such teleost- and cetacean-associated *Pasteurellaceae*, but collectively segregates them from examined *Pasteurellaceae* representatives from terrestrial hosts (Figure 5). The cetacean samples had been collected from humpback whale skin (Apprill et al., 2011), and from the oral cavities of harbor porpoise or either of three oceanic dolphin species (Bik et al., 2016; Godoy-Vitorino et al., 2017; Soares-Castro et al., 2019). These are notably all indigenous to various parts of the north-eastern Atlantic region, where they prey naturally upon pelagic fish.

Historically, the *Pasteurellaceae* family has primarily been associated with mucosal membranes of terrestrial and aquatic mammals (Christensen et al., 2014), which arguably makes the connection of *P. skyensis* and “*P. atlantica*” to cold-blooded fish somewhat peculiar. However, and although upper cardinal growth temperatures seem to vary, all isolates of these two bacterial species tested in our laboratory grow aerobically at temperatures above 30^o^C, and some as high as 37^o^C (unpublished data). These temperatures are consistent with those known to occur on the externally exposed mucosal surfaces of marine mammals (Melero et al., 2015). If pasteurellosis in farmed salmon were in fact linked to infection spillover from cetaceans and/or other marine mammals, however, it would appear that all such historic “host jumps” prior to ca. 2017 have represented epidemiological dead ends, with only limited onward transmission between salmon stocks (Figure 2). This is in contrast to pasteurellosis caused by “*P. atlantica* gv. *cyclopteri*”, which, as mentioned, presents as an endemic and widely disseminated lumpfish pathogen, while “*P. atlantica* gv. *salmonicida*” Pas-4 in Norway (and possibly *P. skyensis* Ps-3 in Scotland) may be on the verge of establishing endemism in salmon aquaculture.

## Conclusion

In conclusion, this study connects pasteurellosis outbreaks amongst sea-farmed fish in Norway and Scotland exclusively with two *bona fide Pasteurellaceae* species presently recognized as *Pasteurella skyensis* and “*Pasteurella atlantica*”. Their current placement within genus *Pasteurella* is, however, highly questionable and the results presented here suggest that they factually belong within the genus *Phocoenobacter*. A number of genetically discrete lineages further exist within each species, and these are also distinguishable based on their host- and/or spatiotemporal associations. Two specific lineages appear to have caused nearly all recent pasteurellosis outbreaks in Norwegian and Scottish farmed salmon, while a third is restricted to lumpfish. Moreover, the very close relatedness of *P. skyensis* and “*P. atlantica*” with *Pasteurellaceae* found worldwide on the mucosal surfaces of various cetacean species supports, but does not prove, marine mammals as a possible reservoir.

## Data availability statement

All datasets presented in this study are accessible via public repositories. The 84 genome assemblies generated as part of this study are available through NCBI under BioProject no. PRJNA727067 (https://www.ncbi.nlm.nih.gov/bioproject/PRJNA727067).

## Author contributions

SG, DC, KL, and HN were the main contributors in regard to conception and design of the study. SG, DC, AO, CÅ, SS, FM, TB, and HN contributed to the collection of bacterial samples and metadata. SG, BS, and KL had main responsibility for the bioinformatics analyses. SG in close collaboration with DC had main responsibility for drafting the manuscript, while all other authors made constructive contributions toward its finalization. All authors contributed to the article and approved the submitted version.
