# Supplementary figures and images for: Phylogeography and host specificity of Pasteurellaceae pathogenic to sea-farmed fish in the north-east Atlantic
Source: Front Microbiol. 2023 Sep 22;14:1236290. doi: 10.3389/fmicb.2023.1236290 (PMC10556747; doi:10.3389/fmicb.2023.1236290)

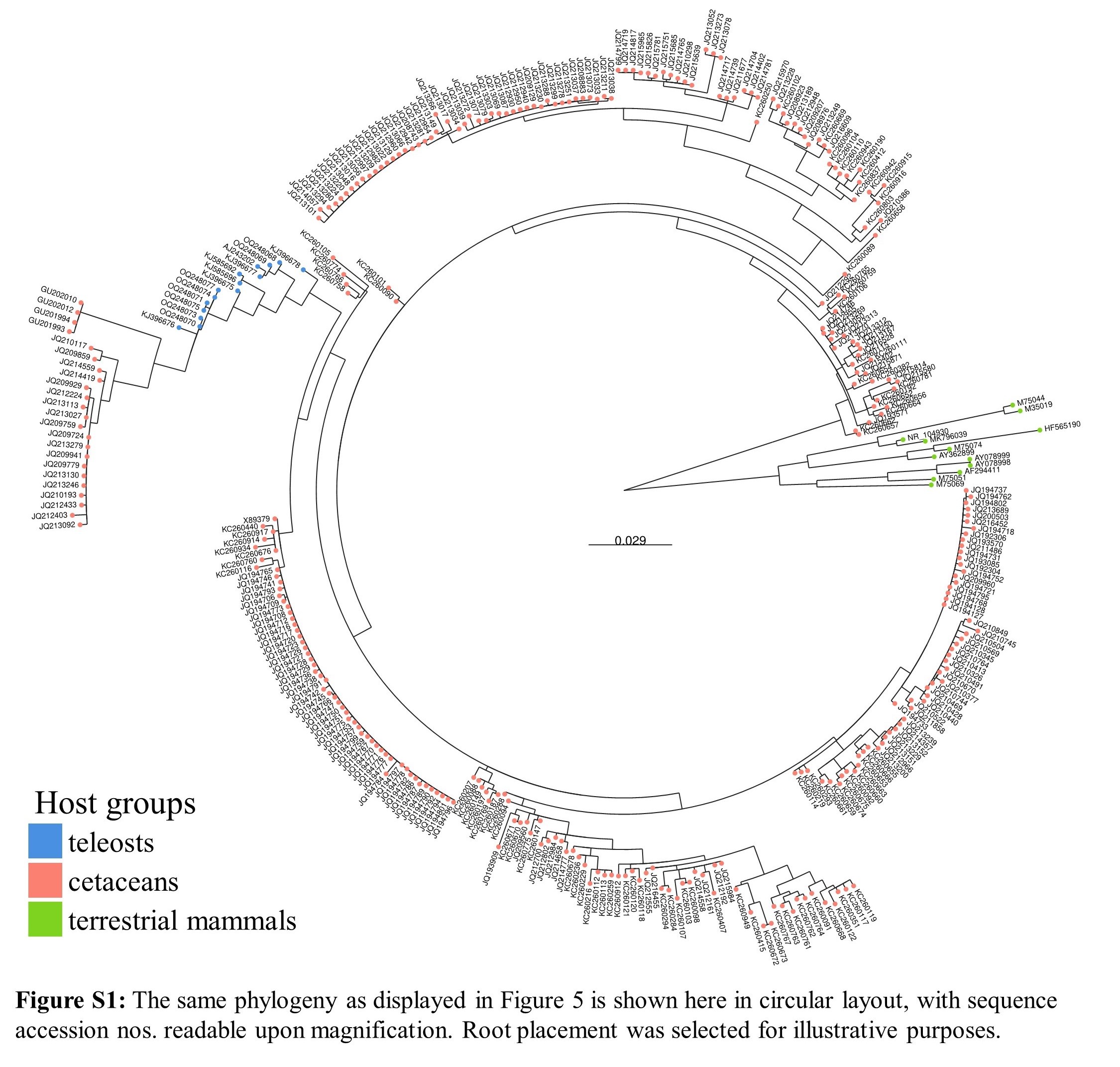

Supplement: Supplementary file 3 [file Image_1.jpg]
